# ACLS4 could be a potential therapeutic target for severe acute pancreatitis

**DOI:** 10.1038/s41598-024-63898-9

**Published:** 2024-06-12

**Authors:** Feng Guo, Yunkun Lu, Lijun Du, Xiuliu Guo, Jinyan Xie, Xiujun Cai

**Affiliations:** 1 Department of Critical Care Medicine, Sir Run Run Shaw Hospital, Zhejiang University School of Medicine, Hangzhou, 310016 People’s Republic of China; 2 Department of General Surgery, Sir Run Run Shaw Hospital, Zhejiang University School of Medicine, Hangzhou, People’s Republic of China; 3 Key Laboratory of Laparoscopic Technology of Zhejiang Province, Department of General Surgery, Sir Run-Run Shaw Hospital, Zhejiang University School of Medicine, 310016 Hangzhou, People’s Republic of China

**Keywords:** Acute pancreatitis, Transcriptome, ACSL4, Inflammation, Gene regulatory networks, Pancreatic disease

## Abstract

Acute pancreatitis (AP) is currently among the most prevalent digestive diseases. The pathogenesis of AP remains elusive, and there is no specific treatment. Therefore, identifying novel therapeutic targets is imperative for effective management and prevention of AP. In this study, we conducted a comprehensive transcriptomic analysis of peripheral blood from patients with AP and the pancreatic tissue from a mouse model of AP. Our analyses revealed that mouse model of AP exhibited a higher enrichment of mitogen-activated protein kinase signaling, endocytosis, apoptosis and tight junction pathways than the control. Subsequent weighted gene co-expression network analysis identified 15 gene modules, containing between 50 and 1000 genes each, which demonstrated significant correlations within samples from patients with AP. Further screening identified four genes (ACSL4, GALNT3, WSB1, and IL1R1) that were significantly upregulated in severe acute pancreatitis (SAP) in both human and mouse samples. In mouse models of SAP, ACSL4 was significantly upregulated in the pancreas, whereas GALNT3, WSB1, and IL1R1 were not. Lastly, we found that a commercially available ACSL4 inhibitor, PRGL493, markedly reduced IL-6 and TNFα expression, alleviated pancreatic edema and necrosis, and diminished the infiltration of inflammatory cells. In conclusion, this study comprehensively depicts the key genes and signaling pathways implicated in AP and suggests the potential of ACSL4 as a novel therapeutic target for SAP. These findings provide valuable insights for further exploration of therapeutic strategies for SAP.

## Introduction

Acute pancreatitis (AP), a prevalent disease of the digestive system, has seen an increasing incidence over recent years^1^. AP is classified into mild acute pancreatitis (MAP) and severe acute pancreatitis (SAP) based on the severity of inflammation. MAP is characterized primarily by pancreatic interstitial edema, with low mortality rate. However, 15–25% of MAP cases may escalate into SAP, which is associated with sustained organ failure and a mortality rate between 36 and 50%, thereby posing an ignificant public health challenge^1–3^.

In the early stages of AP, damaged pancreatic acinar cells release a large number of inflammatory mediators, which recruit immune cells such as macrophages and neutrophils to the affected site and amplify the inflammatory response. This series of reactions will trigger the induction of systemic inflammatory response syndrome (SIRS)^4^. Prolonged SIRS can result in substantial pancreatic necrosis and subsequently affect multiple organs (e.g., lungs, liver, kidneys, and intestines), resulting in multi-organ failure and ultimately death^5^. Effective treatment for SAP is therefore urgently needed.

Recent research has focused on identifying therapeutic targets in the inflammatory cascade of AP. Key among these is the transcription factor NF-kB, which is activated throughout the progression of AP, during which the inflammatory mediators released by damaged acinar cells amplify local and systemic inflammatory responses^6^. NF-kB regulates inflammation by promoting the transcription of inflammatory cytokines, chemokines, and adhesion molecules and managing cell proliferation, apoptosis, morphogenesis, and differentiation^7,8^. Therefore, NF-kB may be a crucial therapeutic target to control immune reactions and slow down the unfolding of AP in early stages. In mouse models of AP, acinar-specific deletion of RelA, which is a NF-kB p65 subunit, significantly increases the disease severity^9^. Activation of NF-kB in pancreatic macrophages or bone marrow-derived macrophages stimulates the release of cytokines and chemokines, leading to systemic inflammation and the generation of a “cytokine storm”^10^. This indicates that molecules such as NF-kB have cell type-specific effects when treated as therapeutic targets.

In this study, we conducted a comprehensive analysis of the peripheral blood transcriptome of patients with AP and the pancreatic tissue transcriptome in a mouse model of AP. Our findings elucidated critical signaling pathways involved in the progression of AP and identified several key target genes, including ACSL4, as a potential therapeutic target and PRGL493, as a potential drug for the treatment of AP.

## Results

### Transcriptome analysis of acute pancreatitis mouse model

**Figure 1:**
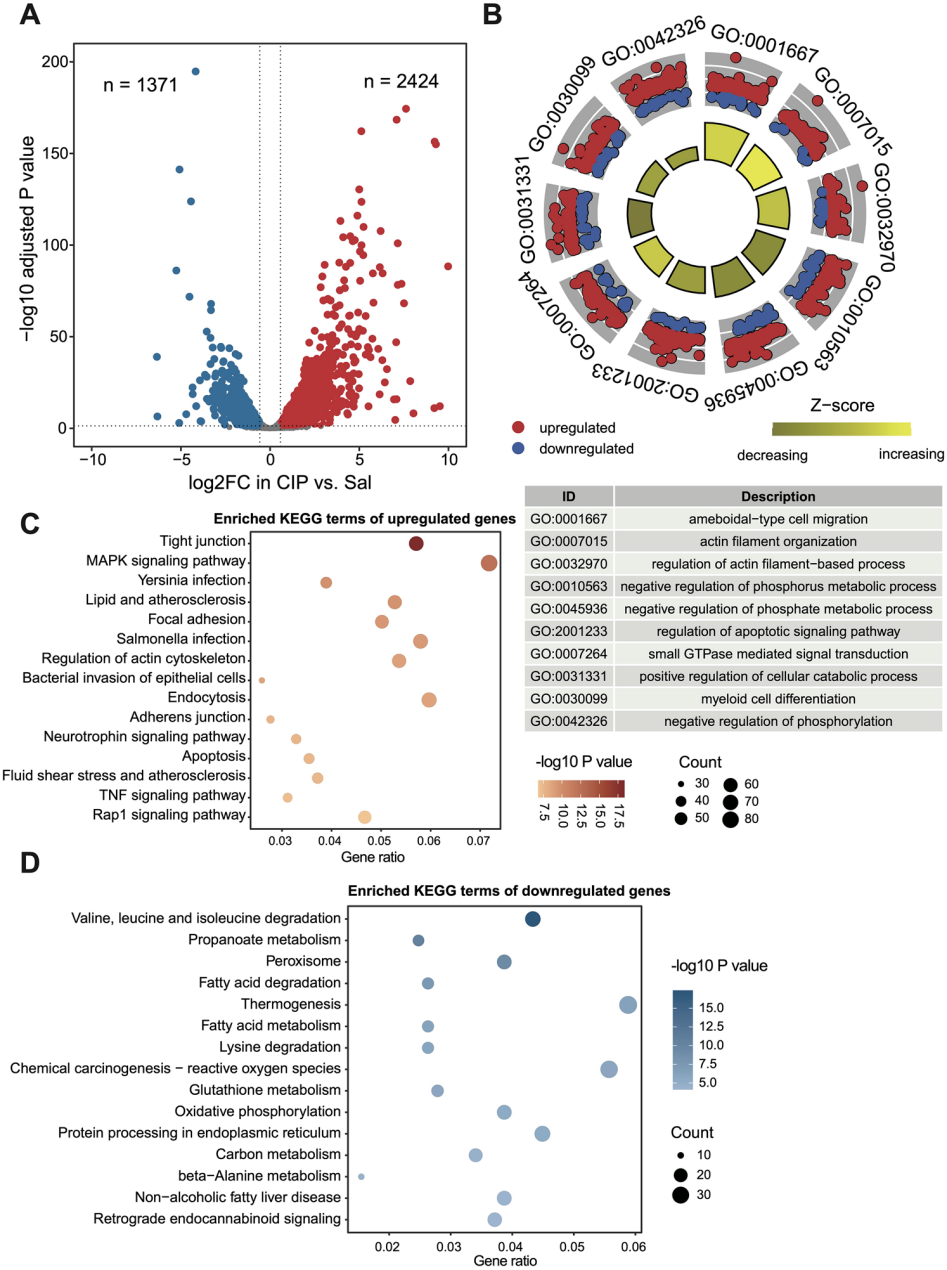
Transcriptome analysis of acute pancreatitis mouse model. (**A**) Volcano plot comparing global gene expression patterns between CIP and Sal treatment mice. The red dots (n = 2424) and blue dots (n = 1371) represent upregulated (FC > 1.5) and downregulated (FC < 0.67) differentially expressed genes with statistical significance (adjusted *P* value < 0.05), respectively. (**B**) Gene Ontology (GO) enrichment circle plot of differentially expressed genes in CIP versus Sal treatment mice. (**C**,**D**) Bubble plots of KEGG pathways^32^ enriched in upregulated (**C**) and downregulated (**D**) genes in CIP versus Sal treatment mice. Cycle size represents the gene numbers in each pathway, color gradients represent the adjusted *P* value of each pathway.

To explore potential therapeutic targets for acute pancreatitis, we initially downloaded transcriptome data of pancreatic tissues from a group of cerulein-induced acute pancreatitis mouse models and their control group from the GEO database (GSE102675). Differential expression analysis between the acute pancreatitis and control mice revealed 2424 upregulated genes (log_2_ fold change > 0.585, adjusted *P* value < 0.05) and 1371 downregulated genes (log_2_ fold change < − 0.585, adjusted *P* value < 0.05) (Fig. 1A). Gene Ontology (GO) enrichment analysis indicated that differentially expressed genes were mainly involved in terms such as ameboidal-type cell migration, actin filament organization, and negative regulation of phosphate metabolic process (Fig. 1B). Similarly, Kyoto Encyclopedia of Genes and Genomes (KEGG) pathway enrichment analysis revealed upregulated genes in pathways like tight junction, MAPK signaling pathway, endocytosis, apoptosis and TNF signaling pathway, while downregulated genes were enriched in various metabolic pathways, such as thermogenesis, protein processing in endoplasmic reticulum, valine, leucine and isoleucine degradation and peroxisome (Fig. 1C,D). Taken together, acute pancreatitis mouse model exhibited a higher enrichment of MAPK signaling, endocytosis, apoptosis and tight junction than the control.

### Weighted gene co-expression network analysis identifies gene modules associated with severe acute pancreatitis

To predict specific therapeutic targets for severe acute pancreatitis accurately, we analyzed transcriptome data from a group of patients with AP (classified as mild, moderate, and severe) and normal healthy controls in the GEO database (GSE194331). There were 87 patients, including 57 (64.8) with mild, 20 (22.7%) with moderate, and 10 (11.4%) with severe AP, and 32 healthy volunteer controls. The gallstone-related AP patients accounted for 49%, idiopathic AP patients was 29%, alcohol-related AP patients was 16%, and the etiology of remaining patients was miscellaneous. There was no remarkable difference in age, sex, or etiology of AP between different AP groups according to the previous AP study from Maryam et al.^11^.

**Figure 2:**
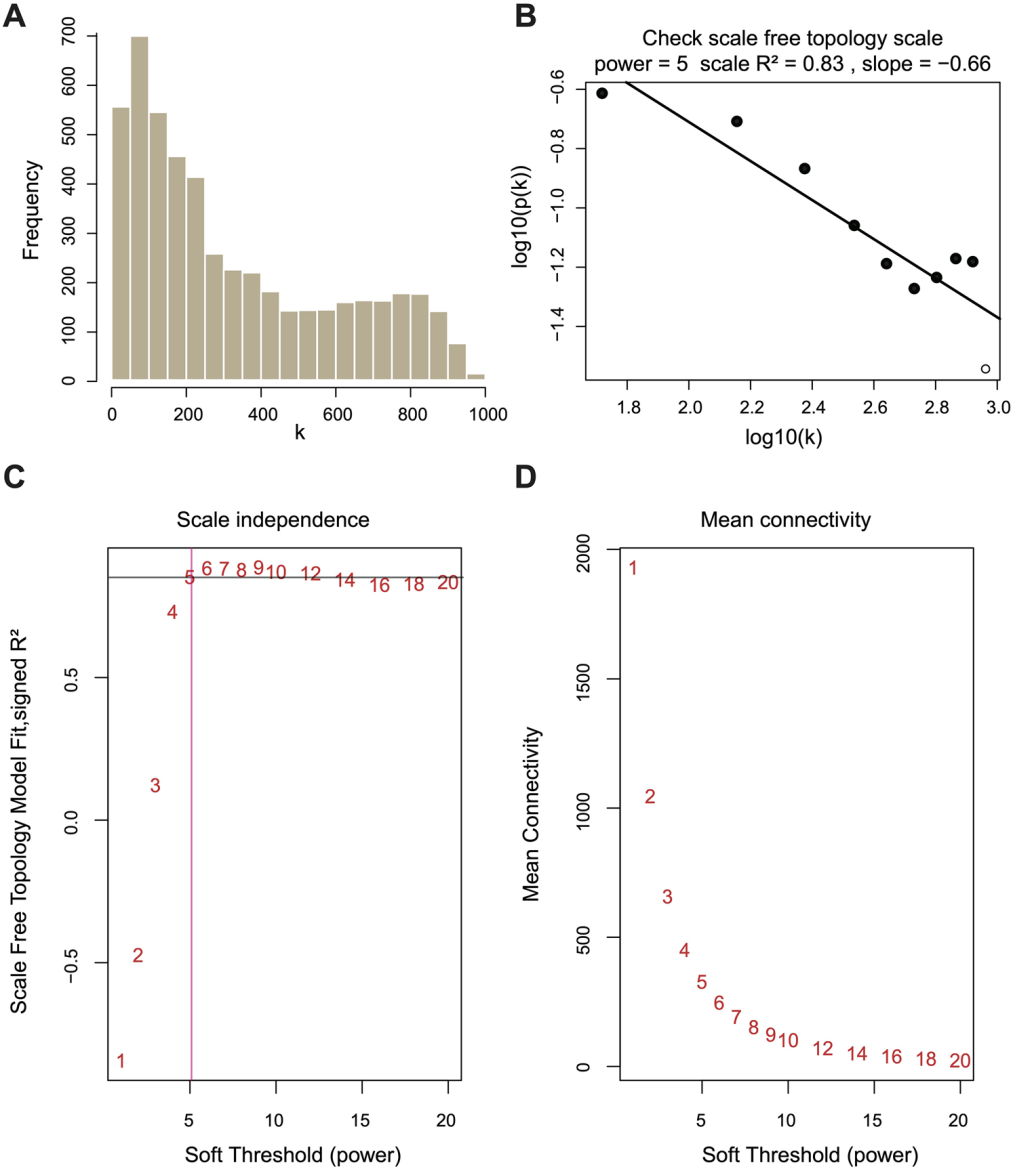
Weighting coefficient β selection. (**A**) Bar diagram of scale-free fit index. (**B**) Scatter plot of different soft-thresholding powers with different mean connectivity. (**C**) Connectivity distribution of nodes with β set as 5. (**D**) The verification of scale-free topology with β set as 5.

We conducted Weighted Gene Co-Expression Network Analysis (WGCNA)^12^ on the top 5000 genes that was the most significant expression differences across all samples. The most important co-expression networks are scale-free networks, such that P(k) ~ k − 1. As the soft threshold (β) increases, the correlation coefficient of the network increases (R^2^ > 0.8) (Fig. 2A), and the mean connectivity of the network subsequently decreases (Fig. 2B). Therefore, β = 5 was selected to construct the co-expression network, such that log(k) was correlated with log[P(k)] (R^2^ = 0.83, slope = − 0.66) (Fig. 2C,D).

**Figure 3:**
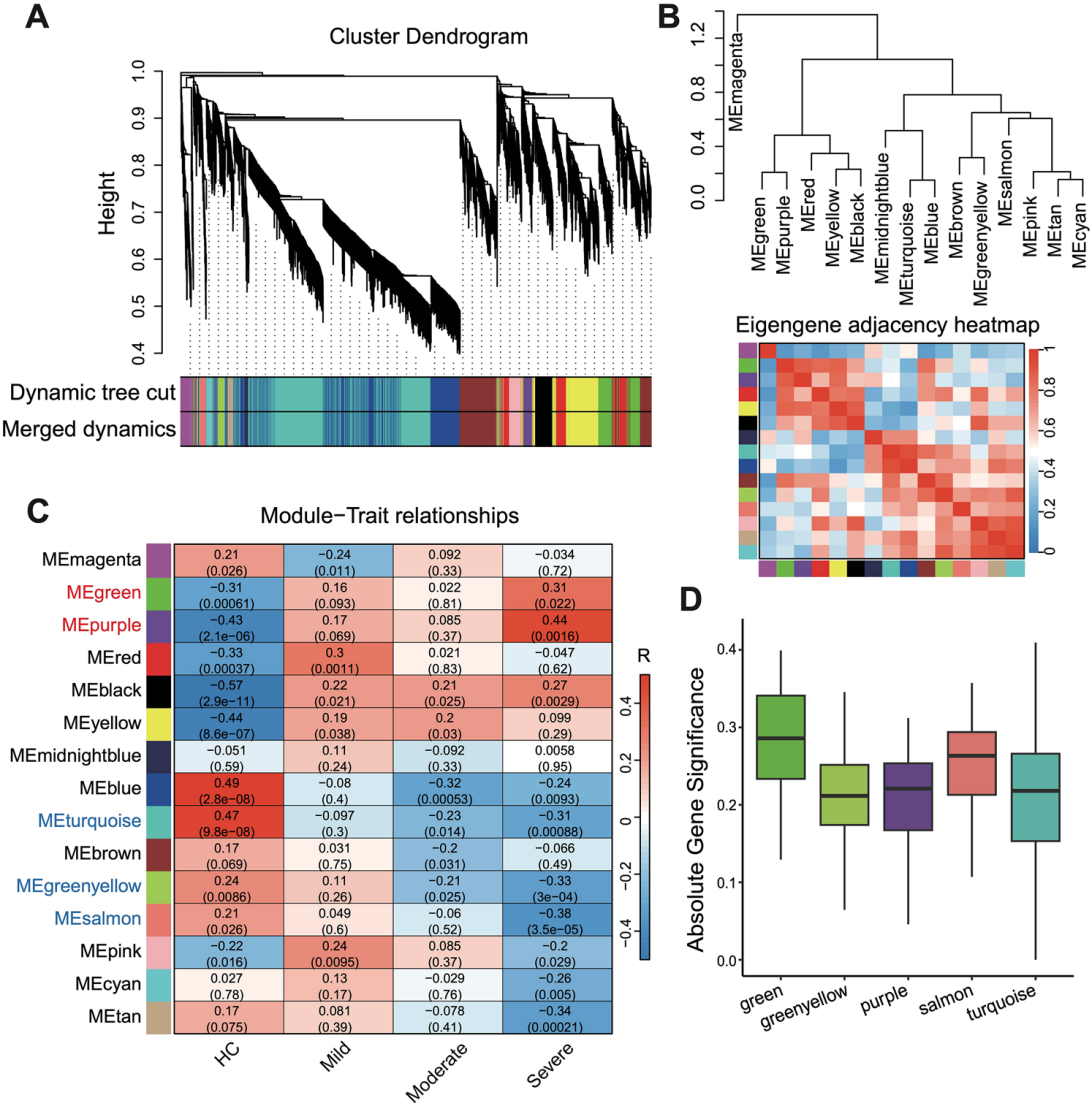
Construction of WGCNA network of acute pancreatitis patient samples. (**A**) Dendrogram of gene topological matrix branch. (**B**) Dendrogram and correlation heatmap of 15 eigengenes. (**C**) Heatmap of correlations between gene modules and clinical types of acute pancreatitis patients. The Pearson correlation coefficient (R) and corresponding *P* value (in parentheses) between different gene modules and clinical traits were shown in the heatmap. Red font represents that the gene modules are positively correlated with severe acute pancreatitis (R > 0.3, *P* < 0.05), and blue font represents that the gene modules are negatively correlated with severe acute pancreatitis (R < − 0.3, *P* < 0.05). (**D**) Boxplots showing the gene significance (GS) for severe acute pancreatitis patients across significantly correlated gene modules.

After selecting the soft threshold, we identified 15 gene modules, each containing 50–1000 genes with significant correlations within acute pancreatitis patient samples (Fig. 3A). Module-feature genes were defined, and Pearson correlation coefficients were calculated to assess the similarity between gene modules, found that the differentiation between gene modules was good, which indicated the correctness of gene modules (Fig. 3B). Module-Trait Relationships (MTRs) were then used to evaluate the correlation between feature genes of each module and the severity levels (mild, moderate, and severe) of acute pancreatitis. Two modules (green and purple) showed significant positive correlation, while three modules (turquoise, green yellow, and salmon) showed significant negative correlation with the severity levels of acute pancreatitis (Fig. 3C). Further analysis revealed that the gene significance (GS) score of the green module was significantly higher, indicating the potential importance of its feature genes in severe acute pancreatitis (Fig. 3D). In summary, WGCNA analysis identified gene modules significantly associated with severe acute pancreatitis, providing potential targets for further investigation.

### Identification of novel therapeutic targets in severe acute pancreatitis

**Figure 4:**
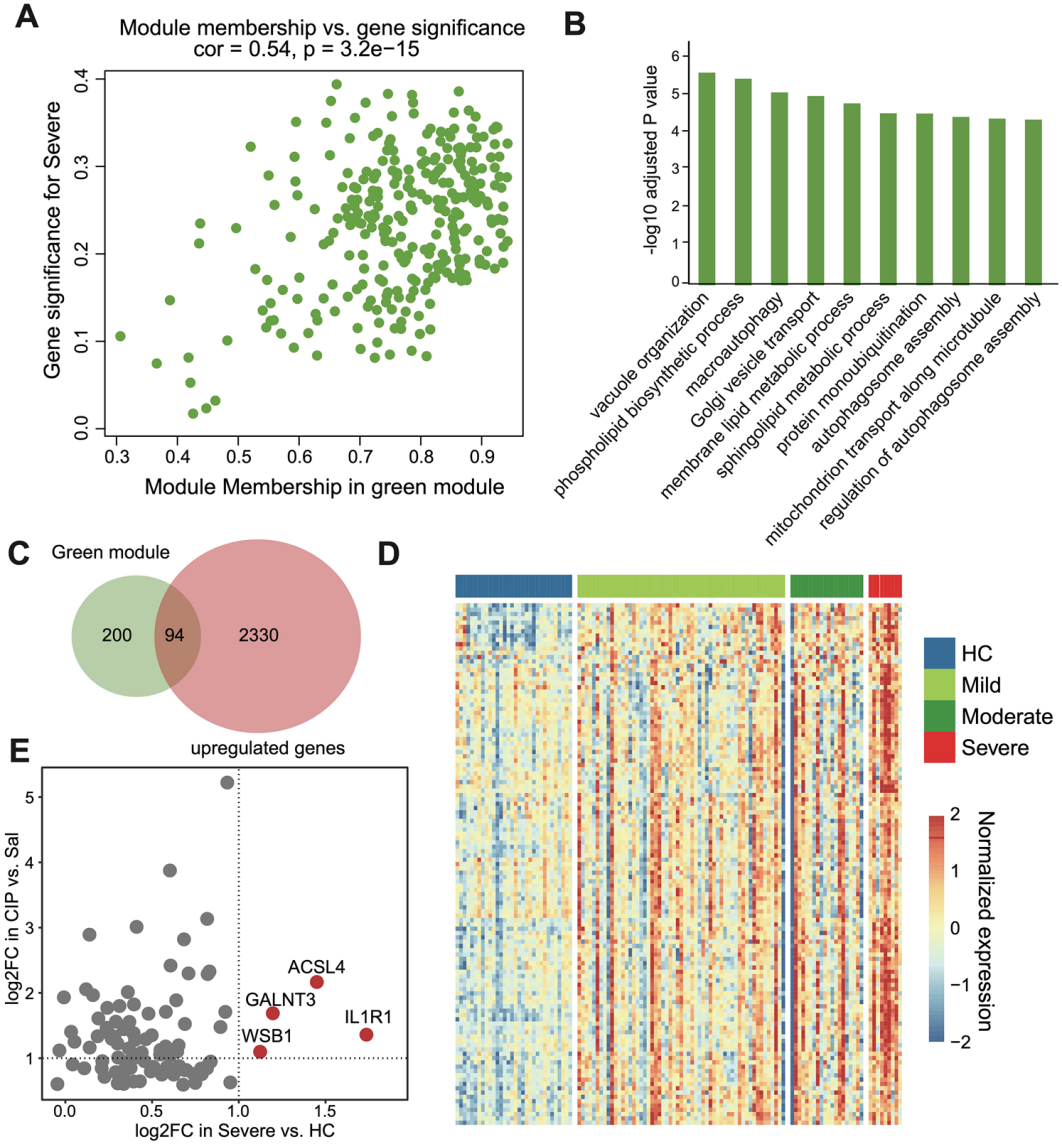
Biomarker identification of severe acute pancreatitis patients. (**A**) Scatter plot of gene significance (GS) for severe patients with module membership (MM) in green module. (**B**) Bar diagram of GO terms enriched in genes of green module. (**C**) Venn diagram of genes in green module and upregulated genes in CIP versus Sal treatment mice. (**D**) Expression heatmap of 94 overlapped genes in acute pancreatitis patient samples. (**E**) Scatter plot showing the log_2_ fold changes of 94 overlapped genes in severe patients versus healthy controls and in CIP versus Sal treatment mice. The genes within both log_2_ fold changes > 1 were highlighted in red dots (ACSL4, GALNT3, WSB1, and IL1R1).

To further explore novel therapeutic targets in severe acute pancreatitis, we focused on the green gene modules. The feature genes in the green module showed a significant positive correlation between gene significance in severe acute pancreatitis and module membership (R = 0.54, *P* value = 3.2e−15) (Fig. 4A). GO enrichment analysis revealed that genes of the green module were associated with functions such as phospholipid biosynthetic process, macro autophagy, Golgi vesicle transport, protein mono-ubiquitination and autophagosome assembly (Fig. 4B). Next, we intersected the genes from the green module with the upregulated genes in the acute pancreatitis mouse model, resulting in 94 common genes (Fig. 4C). These genes exhibited significantly higher expression in acute pancreatitis compared to normal control samples. Moreover, the expression of these genes exhibited a positive correlation with the severity level of acute pancreatitis (Fig. 4D). Further screening identified four genes (*ACSL4*, *GALNT3*, *WSB1*, and *IL1R1*) that were significantly upregulated in severe acute pancreatitis both in human samples and the mouse model (Fig. 4E), suggesting their potential crucial role in acute pancreatitis. Experimental validation of these genes was performed in the subsequent experiments.

### Severe acute pancreatitis upregulates ACSL4

**Figure 5:**
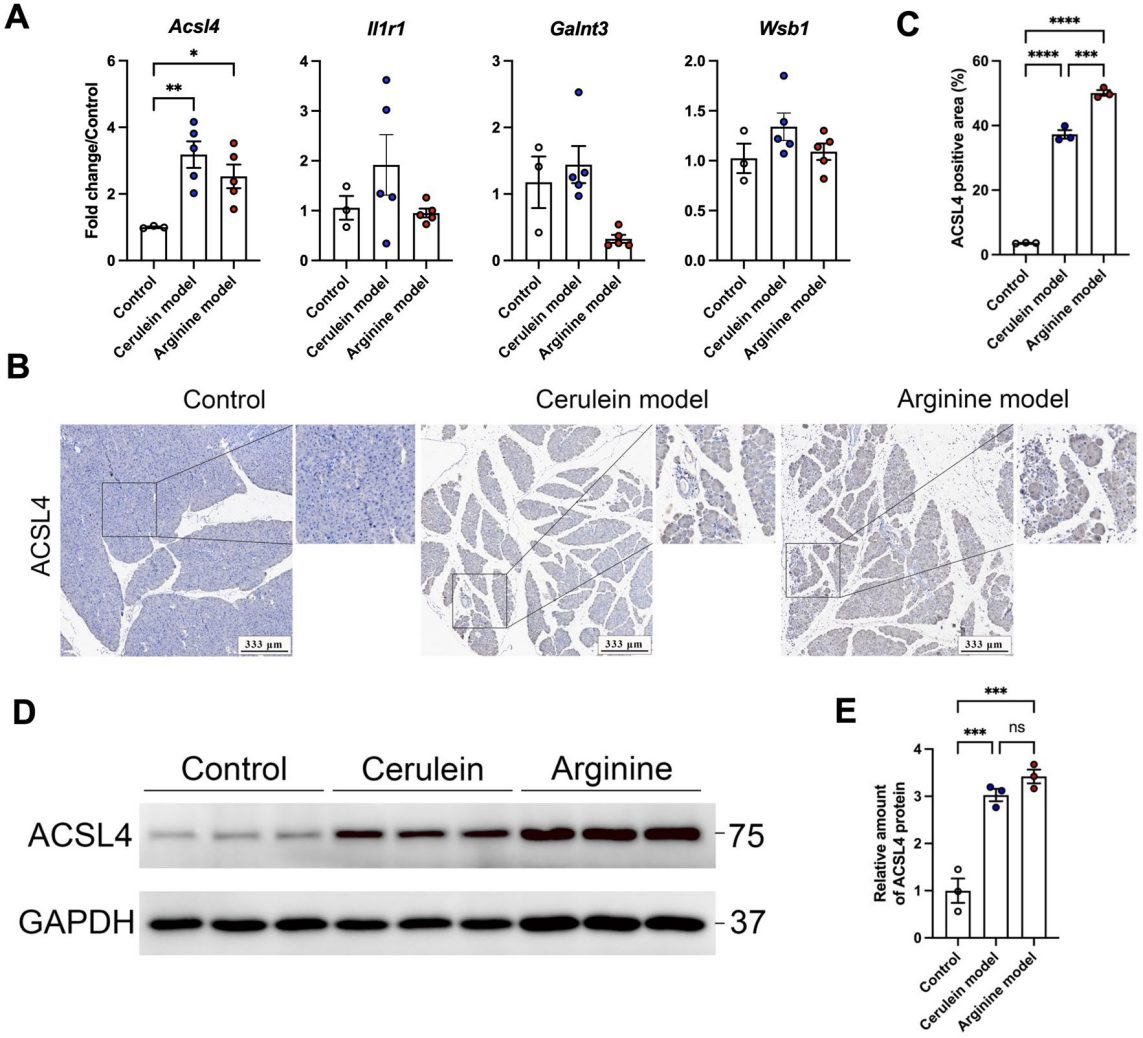
Severe Acute pancreatitis upregulates ACSL4. Mice were injected with cerulein or arginine to induce severe acute pancreatitis. (**A**) qPCR of ACSL4, IL1R1, GALNT3 and WSB1 mRNA levels in pancreas (n = 5). (**B**,**C**) (**B**) IHC images of pancreas section and ACSL4 protein were stained. (**C**) Quantification of ACSL4 positive area (n = 3). (**D**,**E**) (**D**) Western blot of ACSL4 and GAPDH in pancreas. Original blots are presented in Supplementary Fig. 1. (**E**) Quantification of ACSL4 protein. The protein level of ACSL4 was normalized to those of GAPDH (n = 3). *P* values were determined by unpaired two-tailed Student’s t-test; *P < 0.05; **P < 0.01; ***P < 0.001; ****P < 0.0001.

Since the severe acute pancreatitis has a high mortality rate and is more difficult to prevent and cure than mild acute pancreatitis, we first used cerulein and L-arginine to establish a severe acute pancreatitis model in mice, which used to verify the expression of four key genes screened previously (Fig. 4E). The results showed that compared to the control group, the transcription level of ACSL4 in pancreas tissues of cerulein- and arginine-induced severe acute pancreatitis models significantly increased, while there was no significant difference in GALNT3, WSB1 and IL1R1 (Fig. 5A). We further conducted Immunohistochemistry (IHC) to verify the expression of ACSL4, and the results showed that compared with the control group, ACSL4 showed high expression in pancreatic tissues of cerulein- and arginine-induced severe acute pancreatitis models, and the expression of ACSL4 in arginine model was significantly higher than that of cerulein model (Fig. 5B,C). Western blot further verified the upregulation of ACSL4 in pancreas tissue of mice with severe acute pancreatitis compared to control (Fig. 5D,E). These data indicate that ACSL4 expression is significantly upregulated during the development and progression of severe acute pancreatitis.

### ACSL4 inhibitor PRGL493 improves severe acute pancreatitis in mice

**Figure 6:**
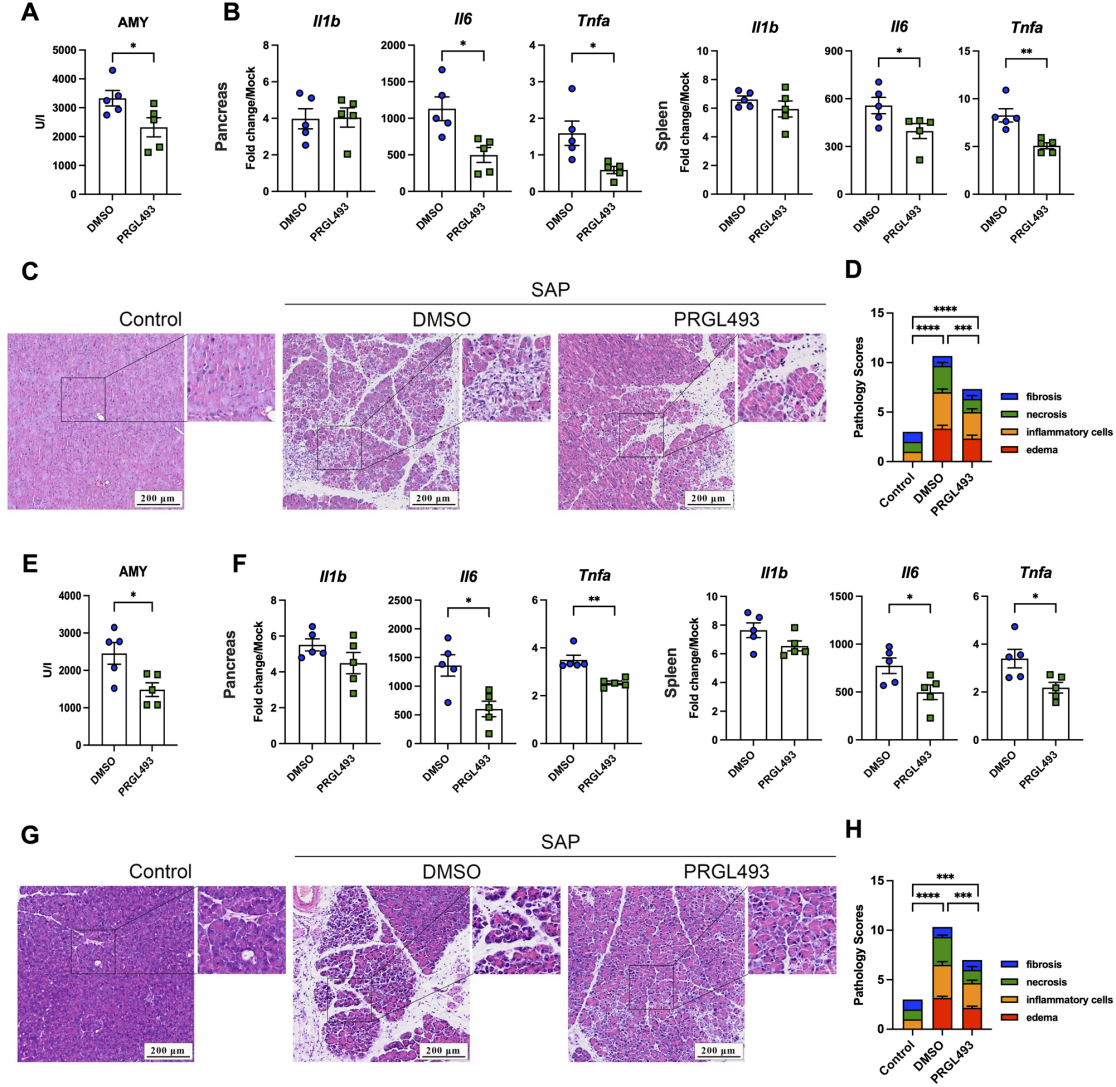
ACSL4 Inhibitor PRGL493 Improves Severe Acute Pancreatitis in Mice. Group of DMSO- and PRGL493-treated mice were injected with arginine to induce severe acute pancreatitis. (**A**) Serum amylase levels (n = 5). (**B**) qPCR of IL-1β, IL-6 and TNF-α mRNA levels in pancreas (left) and spleen (right) (n = 5). (**C**,**D**) representative H&E staining of pancreatic sections with pathology scores (**C**) H.E. staining of pancreas. (**D**) Pancreatic pathology scores. Arginine-induced SAP mice were administered with DMSO or PRGL493. (**E**) Serum amylase levels (n = 5). (**F**) qPCR of ACSL4, IL-1β, IL-6 and TNF-α mRNA levels in pancreas (left) and spleen (right) (n = 5). (**G**,**H**) Representative H&E staining of pancreatic sections with pathology scores (**G**) H.E. staining of pancreas. (**H**) Pancreatic pathology scores. *P* values were determined by unpaired two-tailed Student’s t-test; *P < 0.05; **P < 0.01; ***P < 0.001; ****P < 0.0001.

To further verify whether ACSL4 can be used as a new target for the treatment of severe acute pancreatitis, we administered a commercially available inhibitor of ACSL4, PRGL493, intraperitoneally to mice for 5 consecutive days at a dose of 500 μg/kg body weight, and then injected L-arginine to establish a severe acute pancreatitis model in mice. The results showed that PRGL493 treatment significantly reduced the serum amylase content in mice (Fig. 6A). Subsequently, we detected the expression of inflammatory cytokines and ACSL4 in mouse pancreatic and splenic tissue. The results showed that PRGL493 significantly reduced the expression of ACSL4, IL-6 and TNFα in pancreas and spleen, while it had no significant effect on IL-1β expression (Figs. 6B and S2). Finally, we observed the pathological changes in pancreas of mice, and found that compared with the control group, PRGL493 treatment alleviated pancreatic edema and necrosis and reduced the infiltration of inflammatory cells in the pancreatic tissue of arginine-induced AP model (Fig. 6C,D). These data indicate inhibitor of ACSL4, PRGL493 is of a preventive effect for SAP.

To define the therapeutic effect of ACSL4 inhibitor, L-arginine-induced SAP mice were administered PRGL493 after the onset of SAP. The results show that PRGL493 treatment significantly reduced the serum amylase content in mice (Fig. 6E), and remarkably reduced the expression of IL-6 and TNFα, not IL-1β in pancreas and spleen, (Fig. 6F). Furthermore, we observed that compared with the control group, PRGL493 treatment alleviated pancreatic edema and necrosis and reduced the infiltration of inflammatory cells in the pancreatic tissue of arginine-induced AP model (Fig. 6G,H). Furtherly, we also observed the similar therapeutic effect of PRGL493 in cerulein-induced SAP mice model. In brief, PRGL493 treatment was significantly reduced serum amylase content, local and systemic inflammatory responses, and alleviated pancreatic damage (Fig. S3A–D).

In summary, our data support the potential ACSL4 as a potential therapeutic target for severe acute pancreatitis.

## Discussion

Acute pancreatitis is one of the common diseases in the digestive system, however, the pathogenesis of AP has not been fully elucidated, and there are currently no specific therapeutic agents. Therefore, it is crucial to thoroughly investigate the pathogenesis of AP, explore key therapeutic targets, and identify potential treatment strategies for effective prevention and treatment of AP. In this study, we conducted a comprehensive transcriptomic analysis of an AP mouse model and the peripheral blood of AP patients. We found that in the mouse model of AP, upregulated differentially expressed genes were enriched in the MAPK signaling pathway, which is involved in the regulation of inflammation. Several studies have reported that the inhibitor of p38 MAPK, a key protein in the MAPK signaling pathway, SB203580, significantly reduces the expression of inflammatory factors such as serum amylase, lipase, TNF, and IL-6 in mice^13,14^. Additionally, tight junction pathway is also significantly enriched among the differentially expressed genes identified from the AP mouse model. It has been shown that bacterial translocation and subsequent pancreatic necrotic infection are risk factors for exacerbating AP and late-stage death^15,16^, and bacterial translocation is significantly associated with tight junction^17^. Recent research has found that activated regulatory T cells promote SAP by promoting bacterial translocation to the necrotic area, leading to the deregulation of tight junction genes^18^.

In the transcriptomic analysis of peripheral blood from acute pancreatitis patients, we found that the upregulated genes were mostly related to autophagy (Fig. 4B). The autophagy pathway maintains intracellular homeostasis by regulating the degradation of cytoplasmic components^19^. Dysfunction of the autophagy process has been proven to be a key mechanism in many diseases, including cancer, inflammation, infection, degeneration, and metabolic disorders^20^. Recent studies have indicated that autophagy is one of the early events in acute pancreatitis^21^. Impaired autophagy can promote abnormal activation of zymogen granules, leading to apoptosis and necrosis of exocrine pancreatic cells^21^. Furthermore, it was found that the SQSTM1 (sequestosome 1) in the serum of AP patients is significantly increased, and additional study revealed that the intracellular SQSTM1 protein increased the expression of ACSL4 in a manner dependent on the receptor for advanced glycation end products (AGER), leading to the production of polyunsaturated fatty acids, inducing autophagosome formation, and subsequently causing ferroptosis, exacerbating the course of AP^22^. Interestingly, ACSL4 was identified as the key gene that was commonly induced in both the AP mouse model and peripheral blood of AP patients. In vivo experiments further verified that ACSL4 was significantly upregulated in the pancreatic tissue of the SAP mouse model, and the inhibition of ACSL4 significantly alleviated the SAP-induced inflammatory response and pancreatic injury.

ACSL4 (acyl-CoA synthetase long-chain family member 4) is an enzyme that converts fatty acids into fatty acyl-CoA esters and is closely related to cell membrane lipid synthesis, ferroptosis, and the inflammatory process^23–25^. Recent studies have suggested that overexpression of ACSL4 may lead to excessive activation of the inflammatory response, resulting in inflammatory diseases. In a mouse model of acute kidney injury, the knockout or inhibition of ACSL4 significantly reduced the expression of inflammatory factors such as IL-6 and suppressed ferroptosis, thereby protecting mice from acute kidney injury^26,27^. In this study, we found high expression of ACSL4 in the pancreatic tissue of different AP mouse models, and the inhibition of ACSL4 expression by PRGL493 significantly alleviated the inflammatory response and pancreatic injury induced by SAP in mice (Fig. 6). This may be related to the overexpression of ACSL4 leading to the production of a large amount of polyunsaturated fatty acids, inducing autophagy and ferroptosis^22^. In addition, the activity of ACSL4 is closely related to intracellular lipid metabolism, particularly the lipoxygenase (LOX) pathway. ACSL4-induced lipid metabolites, such as lipid peroxides, can also regulate the inflammatory signaling pathways^28–30^. Moreover, lipid oxidation products (such as 4-HNR and oxPLs) can directly activate Toll-like receptor 4 (TLR4), and the activation of TLR4 induces inflammatory cytokines via NF-kB signaling^31^. Therefore, we postulated that ACSL4-dependent ferroptosis contributes to aberrant intracellular lipid metabolism in acinar cells, leading to the release of a substantial quantity of lipid oxidation products from damaged acinar cells. This triggers TLR4 activation on macrophages, inducing NF-kB-mediated massive production of IL6 and exacerbating pancreatitis.

ACSL4 plays an important role in the inflammatory response during the pathogenesis of acute pancreatitis, and ACLS4-related inhibitor (PRGL493) exhibited the preventive and therapeutic effect to acute pancreatitis (Fig. 6). The potential application of PRGL493 in the prevention and management of acute pancreatitis, including reducing its severity and mortality rates, is envisaged for future clinical use. Our study indicates the potential of targeting ACSL4 for the control of the extent of the inflammatory response, providing a new avenue for the treatment of acute pancreatitis. However, further studies are needed to understand the exact mechanisms and regulatory pathways of ACSL4 in acute pancreatitis.

## Materials and methods

### Ethics statement

The experimental protocols were approved by the Institutional Animal Care and Use Committee of Zhejiang University (No. 117113). All methods were carried out in accordance with relevant guidelines and regulations. This study was conducted as recommended by the ARRIVE guidelines.

### Mice

C57BL/6 J mice were purchased from the Model Animal Research Center of Nanjing University (Nanjing, China). All studies were performed using mice at 6-weeks-of-age that weighed 20 ± 5 g. All mice were housed in a sterile environment with an average temperature of 22 °C and a standard 12 h/12 h light/dark cycle (7:00 am–7:00 pm) in the animal experimentation center of Zhejiang University.

### Induction of different severe acute pancreatitis mouse models

For cerulein-induced severe acute pancreatitis (SAP) model, group of mice were given 7 times intraperitoneal (i.p.) injections of cerulein (50 μg/kg, MCE) with a 1 h interval between injection and a single i.p. injection of LPS (10 mg/kg, sigma) after the last injection of cerulein. Then samples were collected at 12 h after the initial injection of cerulein. For PRGL493 treatment, group of mice were given 2 times intraperitoneal (i.p.) injections of PRGL493 (500 μg/kg, GLPBIO) at 3- and 6-h-post after the first injection of cerulein.

For L-arginine-induced SAP model, group of mice were given 2 hourly i.p. injection with 10% L-arginine (4 g/kg, pH = 7). Mice were received LPS (10 mg/kg) by i.p. at 3 days and samples were collected at 4 h after the initial injection of LPS.

For PRGL493 pretreatment, group of mice were administered PRGL493 via i.p. injection for 5 days, and then constructed L-arginine-induced SAP model.

For PRGL493 therapy experiment, mice were administered 2 hourly i.p. injection with 10% L-arginine (4 g/kg, pH = 7), then administered PRGL493 via i.p. injection for 3 days. Mice were received LPS (10 mg/kg) by i.p. at 3 days and samples were collected at 4 h after the initial injection of LPS.

At the end of all experiments, animals were euthanized followed by immediate blood and pancreas samples collection for further analyses. This protocol consulted the American Veterinary Medical Association (AVMA) Guidelines for the Euthanasia of Animals (2020).

### Western blots

Tissues were homogenized and lysed with RIPA lysis buffer (Beyotime). The lysates were subjected to 10% SDS–polyacrylamide gel electrophoresis and then transferred to polyvinylidene difluoride membranes (Millipore). Proteins were further incubated with the ACSL4 and GAPDH (Proteintech) antibodies and then horseradish peroxidase-conjugated secondary antibodies. Protein bands were probed using an enhanced chemiluminescence kit (Vazyme) with a ChemiDoc Touch Gel Imaging System (Bio-Rad).

### Tissue histology and staining

Pancreas were soaked in 4% paraformaldehyde solution were dehydrated, embedded in paraffin, cut into 4-μm thick sections, and stained with hematoxylin and eosin (HE) using standard procedures.

Deparaffinized pancreas sections were blocked with 10% normal goat serum for 30 min and incubated with anti-ACSL4 (Proteintech) polyclonal antibody overnight at 4 °C. Sections were incubated with peroxidase-conjugated anti-rabbit secondary antibody for 30 min at room temperature. Finally, the sections were incubated with 3,3′-diaminobenzidine solution at room temperature for 1 min. Microscopy was performed, and images were collected by microscopy.

### Cytokine expression analysis

Total RNA from bead-homogenized tissue samples was extracted using TRIzol reagent (Invitrogen) following the manufacturer’s instructions. The cytokine level was determined by quantitative PCR with reverse transcription using the HiScript II One Step qRT–PCR SYBR Green Kit (Vazyme). The following primers were used: IL1R1, forward, GTGCTACTGGGGCTCATTTGT; reverse, GGAGTAAGAGGACACTTGCGAAT. WSB1, forward, ACGAGAAAGAGATCGTGAGATCA; reverse, AGCAAAAGCAACCGTCCAGTT. ACSL4, forward, CTCACCATTATATTGCTGCCTGT; reverse, TCTCTTTGCCATAGCGTTTTTCT. GALNT3, forward, TGCAAATAGGAGCGCCCATTA; reverse, GGCGATCAAAAACCGGCTTC. GAPDH, forward, GCCTTCCGTGTTCCTACCC; reverse, CCCTCAGATGCCTGCTTCAC.

### RNA-seq data analysis

All the previously published RNA-seq datasets were downloaded from the NCBI Gene Expression Omnibus (GEO) database. The accession number of mouse model of acute pancreatitis is GSE102675, the accession number of acute pancreatitis patient samples is GSE194331.

The raw RNA-seq data was trimmed by fastp (v0.20.1) to remove adapter sequences and low-quality reads, and the clean reads were aligned to the mouse genome (mm10) and the human genome (hg38) using HISAT2 (v2.1.0) with the default parameter settings. Gene expression based on the GENCODE vM25 (mouse) and v34 (human) annotations was determined using stringtie (v2.0) with the default parameter settings. Fragments per Kilobase per Million (FPKM) was calculated for representing the expression of each gene. The differential expression analysis was performed by R package DESeq2 (v1.40.2) from the Bioconductor project, and the thresholds of significantly differentially expressed genes (DEGs) were the absolute value of log_2_ fold change ≥ 0.585 and adjusted *P* value < 0.05.

### GO and KEGG pathway enrichment analyses

The GO and KEGG pathway^32^ enrichment analyses in current study were done by R package clusterProfiler (v4.8.1)^33^ from the Bioconductor project. Adjusted *P* value < 0.05 was considered as statistically significant.

### Weighted gene co-expression network analysis (WGCNA)

The weighted gene co-expression network analysis was done according to the protocols of R package WGCNA^12^ (v1.72-1) from the Bioconductor project. First, a pairwise Pearson correlation coefficients matrix to measure the gene–gene similarity of all selected genes across the samples was created based on the normalized gene expression profile. A power adjacency function was performed to transform the similarity matrix into an adjacency matrix, and the adjacency matrix encodes the connection strengths of pairwise nodes in the gene co-expression network. The power β = 5 was chosen as the best soft threshold based on the scale-free topology criterion to determine a scale-free topology index (R^2^) of 0.83. Secondly, we used the Topological Overlap Measure to detect distinct gene modules. This measure is a robust method to calculate the network interconnectedness based on the average linkage hierarchical clustering. Genes were initially cut by the Dynamic Tree-Cut algorithm and represented as branches of the cluster dendrogram, then module eigengene were identified based on the similarity of branches to summarize gene modules by measuring the first principal component of a given module. Next, we used Module-Trait Relationships to determine the significant correlation between module eigengene and healthy controls, mild, moderate, and severe acute pancreatitis patient samples. The heatmaps in Fig. 3 were generated by “plotEigengeneNetworks” and “labeledHeatmap” functions in WGCNA.

### Statistical analysis

Statistical analyses were performed with Prism GraphPad software v 10.0. Error bars represent standard errors of the means in all figures and *P* values (the cutoff for statistical significance was *P* ≤ 0.05) were determined by One-way ANOVA.

### Supplementary Information

Supplementary Information.

## Data Availability

The data that support the findings of this study are available from the corresponding author upon reasonable request.

## References

[1] Banks PA (2013). Classification of acute pancreatitis–2012: Revision of the Atlanta classification and definitions by international consensus. Gut.

[2] Mederos MA, Reber HA, Girgis MD (2021). Acute pancreatitis: A review. JAMA.

[3] Garg PK, Singh VP (2019). Organ failure due to systemic injury in acute pancreatitis. Gastroenterology.

[4] Lee PJ, Papachristou GI (2019). New insights into acute pancreatitis. Nat. Rev. Gastroenterol. Hepatol..

[5] Xiao AY (2016). Global incidence and mortality of pancreatic diseases: A systematic review, meta-analysis, and meta-regression of population-based cohort studies. Lancet Gastroenterol. Hepatol..

[6] Gukovsky I, Gukovskaya AS, Blinman TA, Zaninovic V, Pandol SJ (1998). Early NF-kappaB activation is associated with hormone-induced pancreatitis. Am. J. Physiol..

[7] Oeckinghaus A, Hayden MS, Ghosh S (2011). Crosstalk in NF-κB signaling pathways. Nat. Immunol..

[8] He G, Karin M (2011). NF-κB and STAT3—Key players in liver inflammation and cancer. Cell Res..

[9] Algül H (2007). Pancreas-specific RelA/p65 truncation increases susceptibility of acini to inflammation-associated cell death following cerulein pancreatitis. J. Clin. Invest..

[10] Sendler M (2018). Cathepsin B-mediated activation of trypsinogen in endocytosing macrophages increases severity of pancreatitis in mice. Gastroenterology.

[11] Nesvaderani M (2022). Gene expression profiling: Identification of novel pathways and potential biomarkers in severe acute pancreatitis. J. Am. Coll. Surg..

[12] Langfelder P, Horvath S (2008). WGCNA: An R package for weighted correlation network analysis. BMC Bioinform..

[13] Zhang J (2021). Inhibition of the p38 MAPK pathway attenuates renal injury in pregnant rats with acute necrotizing pancreatitis. Immunol. Res..

[14] Cao MH (2015). p38 MAPK inhibition alleviates experimental acute pancreatitis in mice. Hepatobiliary Pancreat. Dis. Int..

[15] Liu J, Huang L, Luo M, Xia X (2019). Bacterial translocation in acute pancreatitis. Crit. Rev. Microbiol..

[16] Li XY, He C, Zhu Y, Lu NH (2020). Role of gut microbiota on intestinal barrier function in acute pancreatitis. World J. Gastroenterol..

[17] Meriläinen S (2012). Intestinal bacterial translocation and tight junction structure in acute porcine pancreatitis. Hepatogastroenterology.

[18] Glaubitz J (2023). Activated regulatory T-cells promote duodenal bacterial translocation into necrotic areas in severe acute pancreatitis. Gut.

[19] D’Arcy MS (2019). Cell death: A review of the major forms of apoptosis, necrosis and autophagy. Cell Biol. Int..

[20] Klionsky DJ (2021). Autophagy in major human diseases. Embo J..

[21] Zhang T, Gan Y, Zhu S (2023). Association between autophagy and acute pancreatitis. Front. Genet..

[22] Yang L (2023). Extracellular SQSTM1 exacerbates acute pancreatitis by activating autophagy-dependent ferroptosis. Autophagy.

[23] Doll S (2017). ACSL4 dictates ferroptosis sensitivity by shaping cellular lipid composition. Nat. Chem. Biol..

[24] Yuan H, Li X, Zhang X, Kang R, Tang D (2016). Identification of ACSL4 as a biomarker and contributor of ferroptosis. Biochem. Biophys. Res. Commun..

[25] Kuwata H, Hara S (2019). Role of acyl-CoA synthetase ACSL4 in arachidonic acid metabolism. Prostaglandins Other Lipid Mediat..

[26] Wang Y (2022). ACSL4 deficiency confers protection against ferroptosis-mediated acute kidney injury. Redox Biol..

[27] Tao WH (2022). Dexmedetomidine attenuates ferroptosis-mediated renal ischemia/reperfusion injury and inflammation by inhibiting ACSL4 via α2-AR. Front. Pharmacol..

[28] Zhang HL (2022). PKCβII phosphorylates ACSL4 to amplify lipid peroxidation to induce ferroptosis. Nat. Cell Biol..

[29] Pang Y (2022). Edaravone modulates neuronal GPX4/ACSL4/5-LOX to promote recovery after spinal cord injury. Front. Cell Dev. Biol..

[30] Yamada N (2020). Ferroptosis driven by radical oxidation of n-6 polyunsaturated fatty acids mediates acetaminophen-induced acute liver failure. Cell Death Dis..

[31] Chen X, Kang R, Kroemer G, Tang D (2021). Ferroptosis in infection, inflammation, and immunity. J. Exp. Med..

[32] Kanehisa M, Goto S (2000). KEGG: Kyoto encyclopedia of genes and genomes. Nucleic Acids Res..

[33] Wu T (2021). clusterProfiler 4.0: A universal enrichment tool for interpreting omics data. Innovation.

