# Supplementary material for: ACLS4 could be a potential therapeutic target for severe acute pancreatitis
Source: Sci Rep. 2024 Jun 12;14:13457. doi: 10.1038/s41598-024-63898-9 (PMC11166934; doi:10.1038/s41598-024-63898-9)
Supplement: Supplementary file 1 — Supplementary Information. [file 41598_2024_63898_MOESM1_ESM.pdf]

Supplementary Information

ACLS4 could be a potential therapeutic target for severe acute pancreatitis

Feng Guo1†, Yunkun Lu2†, Lijun Du2, Xiuliu Guo1, Jinyan Xie1*, Xiujun Cai2,3*

[1] Department of Critical Care Medicine, Sir Run Run Shaw Hospital, Zhejiang University School of Medicine, Hangzhou, 310016, P. R. China;

[2] Department of General Surgery, Sir Run Run Shaw Hospital, Zhejiang University School of Medicine, Hangzhou, China;

[3] Key Laboratory of Laparoscopic Technology of Zhejiang Province, Department of General Surgery, Sir Run-Run Shaw Hospital, Zhejiang University School of Medicine, 310016, Hangzhou, China.

†Contributed equally to this study.

*Corresponding author.

Jinyan Xie: xiejinyan1110@zju.edu.cn

Xiujun: srrsh_cxj@zju.edu.cn

Supplementary Figure 1

A

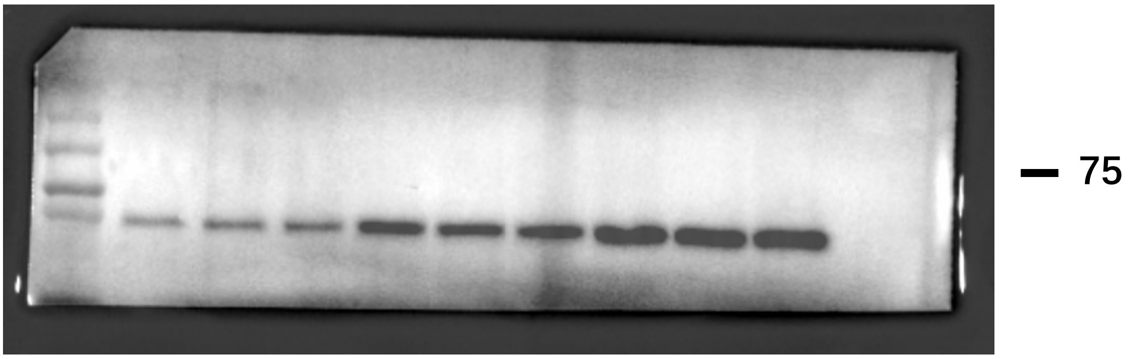

B

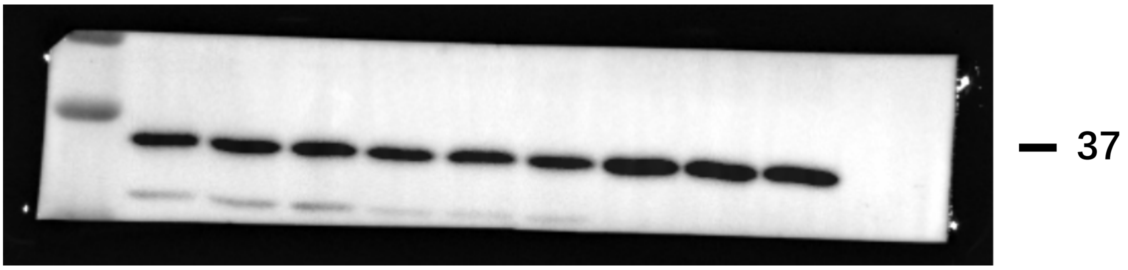

Figure S1. original blots of Figure 5D

A. The original blot of ACSL4 in Figure 5D. B. The original blot of GAPDH in Figure 5D.

Supplementary Figure 2

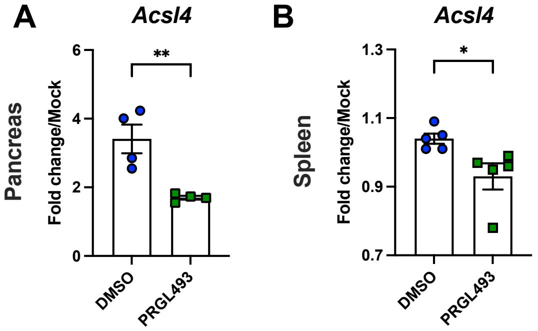

Figure S2. ACSL4 Inhibitor PRGL493 Improves Severe Acute Pancreatitis in Mice.

Group of DMSO- and PRGL493-treated mice were injected with arginine to induce severe acute pancreatitis. (A-B) qPCR of ACSL4mRNA levels in pancreas (A) and spleen (B) (n=5). *P* values were determined by unpaired two-tailed Student's t-test; *: $P < 0.05$; **: $P < 0.01$.

Supplementary Figure 3

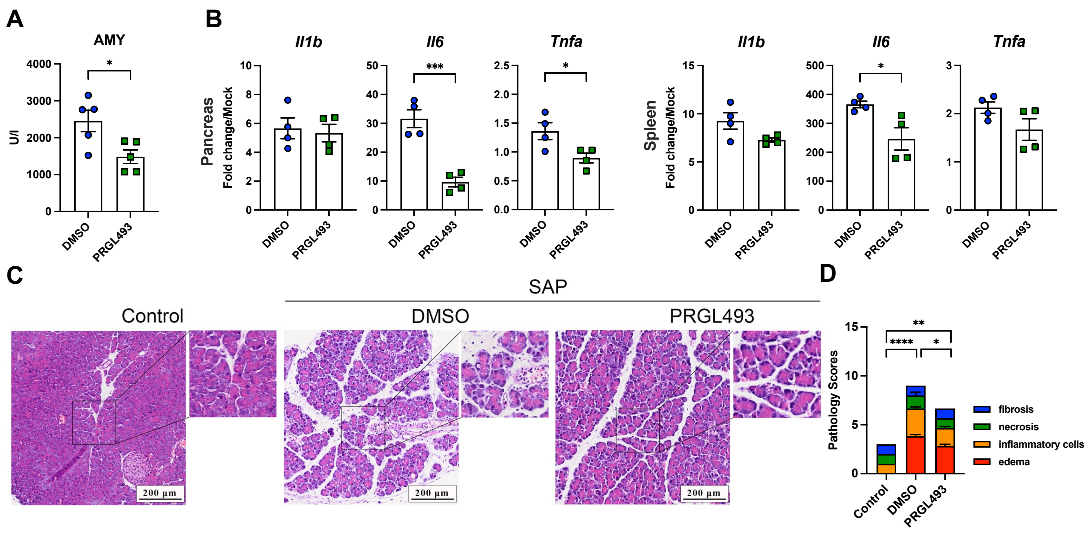

Figure S3. ACSL4 Inhibitor PRGL493 Improves Severe Acute Pancreatitis in Mice.

Cerulein-induced SAP mice were administered with DMSO or RPGL493. (A) serum amylase levels (n=5). (B) qPCR of IL-1β, IL-6 and TNF-α mRNA levels in pancreas (left) and spleen (right) (n=5). (C-D) representative H&E staining of pancreatic sections with pathology scores (C) H.E. staining of pancreas. (D) pancreatic pathology scores. *P* values were determined by unpaired two-tailed Student's t-test; *: $P < 0.05$; **: $P < 0.01$; ***: $P < 0.001$; ****: $P < 0.0001$.
